# Similar response rates and survival with PARP inhibitors for patients with solid tumors harboring somatic versus Germline BRCA mutations: a Meta-analysis and systematic review

**DOI:** 10.1186/s12885-020-06948-5

**Published:** 2020-06-03

**Authors:** Ghulam Rehman Mohyuddin, Muhammad Aziz, Alec Britt, Lee Wade, Weijing Sun, Joaquina Baranda, Raed Al-Rajabi, Anwaar Saeed, Anup Kasi

**Affiliations:** 1 University of Kansas, Kansas City, USA; 2 Department of Internal Medicine, University of Toledo, Toledo, USA; 3 Department of Internal Medicine, University of Kansas, Kansas City, USA; 4 University of Toledo Libraries, Toledo, USA; 5 Division of Medical Oncology, University of Kansas Cancer Center, Kansas City, USA

**Keywords:** PARP inhibitors, BRCA, Somatic, Germline, Meta-analysis

## Abstract

**Background:**

PARP inhibitors (PARPi) have recently been approved for various malignancies based on the results of several clinical trials. However, these trials have mostly recruited patients with germline BRCA mutations, and it is unclear whether PARPi have similar efficacy in patients with somatic BRCA mutations. Our study aimed to determine the efficacy of PARPi in patients with somatic BRCA mutations.

**Methods:**

We performed a meta-analysis comparing overall response rate to PARPi in patients harboring somatic versus germline BRCA mutations. We looked at studies including somatic and germline mutations in BRCA patients that received PARPi.

**Results:**

After screening and removing duplicates, 18 studies met our criteria for including both somatic and germline BRCA mutations. Only 8 studies reported response rates for both somatic and germline BRCA mutations.

In those studies, 24 out of 43 patients with somatic BRCA mutations (55.8%), and 69 out of 157 (43.9%) patients with germline BRCA patients had a response to therapy to PARPi. This difference was not statistically significant (*p* = 0.399).

In all five studies that reported progression-free survival, there was no obvious difference in outcomes between somatic versus germline BRCA patients, however a precise statistical analysis could not be performed.

**Conclusion:**

Our meta-analysis and systematic review of the literature indicates similar response rates of PARPi therapy in patients with somatic and germline BRCA mutations. Investigation of use of PARPi therapy in a broader patient population, and the inclusion of somatic BRCA mutations in further clinical trials is paramount in improving therapeutic options for our patients.

## Background

Since their discovery in 1994 and 1995 respectively, there has been significant clinical interest in the tumor suppressor genes BRCA1 and BRCA2, with particular focus in recent years on targeted therapies for patients with BRCA mutated cancers. The BRCA1-encoded protein is an important DNA damage response protein that interacts with multiple sensor and effector proteins in the DNA repair pathways, and is also involved directly in the repair of double-stranded DNA breaks, and BRCA2 is primarily involved in facilitating homologous recombination repair of DNA damage [1, 2]. Defects in function therefore lead to dysfunctional chromosomal rearrangement and cellular replication. Deleterious germline mutations in the BRCA1 protein have been found to significantly increase the risk of breast cancer and ovarian cancer, up to 72 and 44% respectively by age 80, as well as increased risk of many gastrointestinal, pancreatic and prostate cancers [3, 4]. BRCA2 deleterious mutations confer a similar risk of breast and ovarian cancer, as well as pancreatic cancer, prostate cancer, stomach cancer, and melanoma as well [3, 5]. Somatic BRCA mutations which are present only in the tumor cells, have been reported to be up to 15–30% of all BRCA1/2 mutations, and can be found in various malignancies, such as 3% of breast cancer cases, and over 12% of advanced prostate cancer patients [6–10].

Poly(ADP-ribose) polymerase (PARP) is a nuclear protein that is activated by breaks in DNA single strands, which then further recruits DNA repair proteins with synthesis of poly(ADP-ribose) chains [11]. A class of medications, the PARP inhibitors (PARPi), were developed to specifically target the DNA repair pathways involved with PARP1, as well as the other PARP enzymes. Use of PARPi was found to increase apoptosis of BRCA1 and BRCA2 mutated cells, due to a combination of increased DNA lesions and inability for defective BRCA1 and BRCA2 products to repair the DNA, an example of the genetic concept called synthetic lethality [12, 13]. Multiple PARPi have since been FDA approved for advanced ovarian cancer and/or breast cancer, such as olaparib in 2014, rucaparib in 2016, niraparib in 2017, and talazoparib in 2018 [14]. The study of PARPi has been mostly limited to patients with germline BRCA mutations and hence there is a lack of data comparing response rates to PARPi in patients with somatic versus germline BRCA mutations. Furthermore, there is a concern that somatic BRCA mutations detected in tumor tissue, may be passenger mutations instead of driver mutations, and thus targeting it may not be of utility [15]. In order to better ascertain this, we performed a meta-analysis comparing overall response rate to PARPi in patients harboring a somatic versus germline BRCA mutation. We looked at all published studies including somatic and germline mutations in BRCA patients that received PARPi.

## Methods

### Search strategy

The search strategy was developed and verified by two authors (W.L-S and G.M). The following databases were queried for the purpose of this manuscript: PubMed/Medline, Embase, Cochrane Register of Controlled Trials, and Web of Science Core Collection from inception through November 15th 2019. Medical subject headings and keyword synonyms for the concepts of Somatic mutations, germline mutations, and PARPi were developed on PubMed and translated for querying other databases. The detailed search strategy for Embase is presented as example in supplementary Table 1. Relevant articles were initially screened based on title and abstracts. The articles were finalized for the purpose of this review by two reviewers (G.M and A.K) and the discrepancy was resolved by a third reviewer (M.A).

**Fig. 1:**
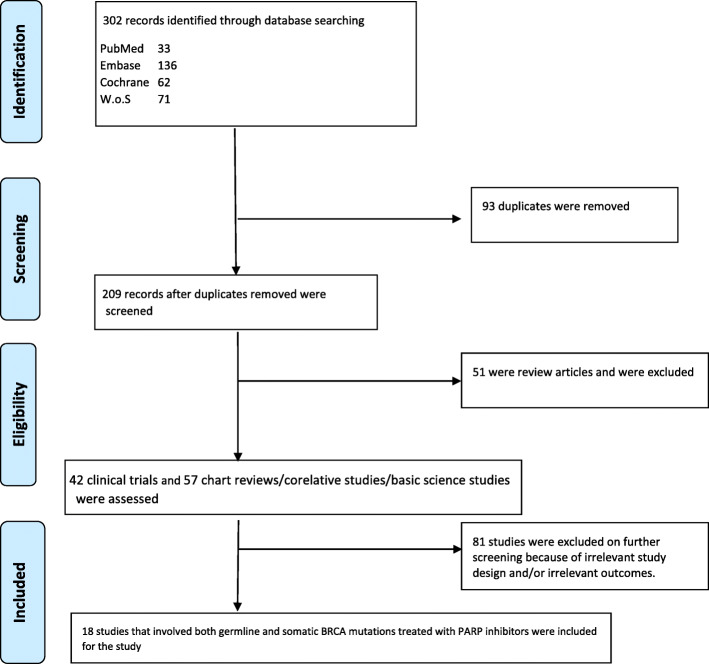
Flow diagram describing our data collection

Preferred Reporting Items for Systematic Reviews and Meta-Analyses (PRISMA) guidelines were adhered to for the purpose of this manuscript [16]. Figure 1 shows search strategy and findings, screening, study selection and exclusion and final analysis.

### Study definitions

Somatic BRCA mutations were defined as either a BRCA1 or BRCA2 mutations present only in the tumor tissue. Germline BRCA mutations were defined as either a BRCA1 or BRCA2 mutation that was present on germ cells, identified by the study authors through genetic testing of non-tumor tissue, such as a blood test. Response rate was defined as a reduction in tumor size per the respective studies using RECIST criteria whenever applicable [17]. Progression-free survival was defined as per the respective studies from the time of initiation of treatment to the onset of progression or death from any cause, defined as per RECIST criteria, whenever applicable [17].

### Inclusion and exclusion criteria

Our study inclusion criteria were all studies (including retrospective studies and clinical trials) that reported use of PARPi in patients with both somatic and germline BRCA patients. Our search strategy was limited to randomized controlled trials (RCT) and cohort studies only. We excluded all other studies including single arm studies, case reports, small case series, editorials, review articles, and perspectives. Our search was not restricted to language or dates. We also included abstracts for the purpose of this review. We only included studies that reported on results for both somatic and germline BRCA mutations in our quantitative analysis, and hence studies that included just patients with somatic mutations, or just germline mutations were not included.

### Data collection

The underlying cancer type, phase/type of study and number/responses of patients with somatic and germline mutations were extracted where applicable. Type of treatment regimen and data of progression-free survival (PFS) was extracted. The data extraction was performed by two reviewers (G.M. and M.A.) and cross verified to resolve any discrepancy.

### Primary and secondary outcomes

Our primary objective was to compare the overall response rate (ORR) with PARPi therapy for patients harboring somatic versus germline BRCA mutations. We also aimed to assess progression-free survival (PFS) data in patients with somatic versus germline BRCA mutations.

### Data synthesis and statistical analysis

We extracted data using Microsoft Excel (Microsoft, Redmond, Washington, United States). Proportional outcomes were pooled using random effects model and DerSimonian-Laird Method. Risk ratio (RR) with 95% confidence interval (CI) was calculated for each outcome. A *p*-value of < 0.05 was considered statistically significant when comparing outcomes. The fixed effect model was utilized as a sensitivity tool. The I2 statistic was used to test for heterogeneity between the studies as defined by Cochrane handbook for systematic reviews. The I2 of values of < 30, 30–60%, 61–75%, and > 75% were suggestive of low, moderate, substantial, and considerable heterogeneity, respectively [18, 19]. [The outcomes were generated using Open Meta Analyst (CEBM, University of Oxford, Oxford, United Kingdom) and Review Manager v5.3 (Cochrane Collaboration, London, United Kingdom)].

### Bias assessment

We used the Cochrane Risk of bias tools for RCTs and Newcastle Ottawa score for cohort studies [18–20]. Publication bias was assessed using funnel plot generated on Review Manager (Cochrane Collaboration, London, United Kingdom).

## Results

Figure 1 highlights our data collection process. 302 studies were identified from database searches. After removal of duplicates, 42 clinical trials and 57 chart reviews/corelative studies/basic science studies were assessed further. 18 studies met our criteria for including both somatic and germline BRCA mutations and were assessed further. Only 8 studies reported ORR for both somatic and germline BRCA mutations.

### Characteristics of included studies

Amongst the 18 studies that we identified that included both somatic and germline BRCA mutations treated with PARPi, 14 studies involved use of PARPi as monotherapy, whereas 4 involved use of PARPi in combination with other therapies. 6 studies evaluated PARPi in a maintenance setting. Olaparib was studied in 7 [21–27], rucaparib in 4 [28–31], niraparib in 3 [32–34], and talazoparib in 2 studies [35, 36] respectively. 10 of the studies were for ovarian cancer patients, 2 for pancreatic cancer, 3 for prostate cancer, 2 for multiple solid tumors, and 1 for cholangiocarcinoma respectively.

Across all 18 studies, a total of 236 patients with somatic BRCA mutations were treated with PARPi, and 1204 patients with germline BRCA mutations were treated with PARPi. When accounting for only the monotherapy PARPi studies, there were 196 somatic and 1044 germline patients.

### Overall response rate data

A total of eight studies described ORR data for both somatic and germline patients separately in either the abstract, manuscript or appendix. Table 1 includes data on these studies.

**Table 1:** Characteristics of studies included in our meta-analysis (mo = months, NA = not available)

Study	Type of Cancer	Type of Drug Used	PARP Combo versus Mono	Male (%)	Average Age	Duration of follow-up	Total number of patients (n)	Somatic (n)	Germline	BRCA 1/2 breakdown	ORR rate for somatic	ORR rate for germline
Dhawan	Multiple	Talazoparib	Combo	83.3%	59 (42–77)	NA	24	1	7	Somatic: 0/1 Germline: 3/4	0/1	3/7
Abida	Prostate	Rucaparib	Monotherapy	100%	71 (49–88)	3.7 mo	85	12	10	NA	5/10	5/10
Konstatintopaulos et al	Ovarian	Olaparib	Combo	0%	60 (55–67)	12 mo	34	3	10	Somatic: 0/3 Germline: 7/5^a^	1/3	3/10
Oza et al	Ovarian	Rucaparib	Monotherapy	0%	59 (33–84)	NA	106	18	88	Overall: 67/41	10/18	47/88
Mateo et al	Prostate	Olaparib	Monotherapy	100%	68 (41–73)	14.4 mo	50	4	3	Overall: 0/7	4/4	3/3
Binder et al	Pancreatic	Rucaparib	Monotherapy	15.8%	61 (35–81)	NA	24	1	16	Somatic: 0/1 Germline: 3/13	1/1	6/16
Schroff et al	Pancreatic	Rucaparib	Monotherapy	57.9%	57 (41–75)	NA	19	3	16	Overall: 4/15	2/3	1/16
Piha Paul et al	Multiple	Talazoparib	Monotherapy	NA	NA	15.8 mo	35	1	7	Na	0/1	1/7

^a^ 2 patients were reported to have both BRCA 1 and 2 germline mutations

**Fig. 2:**
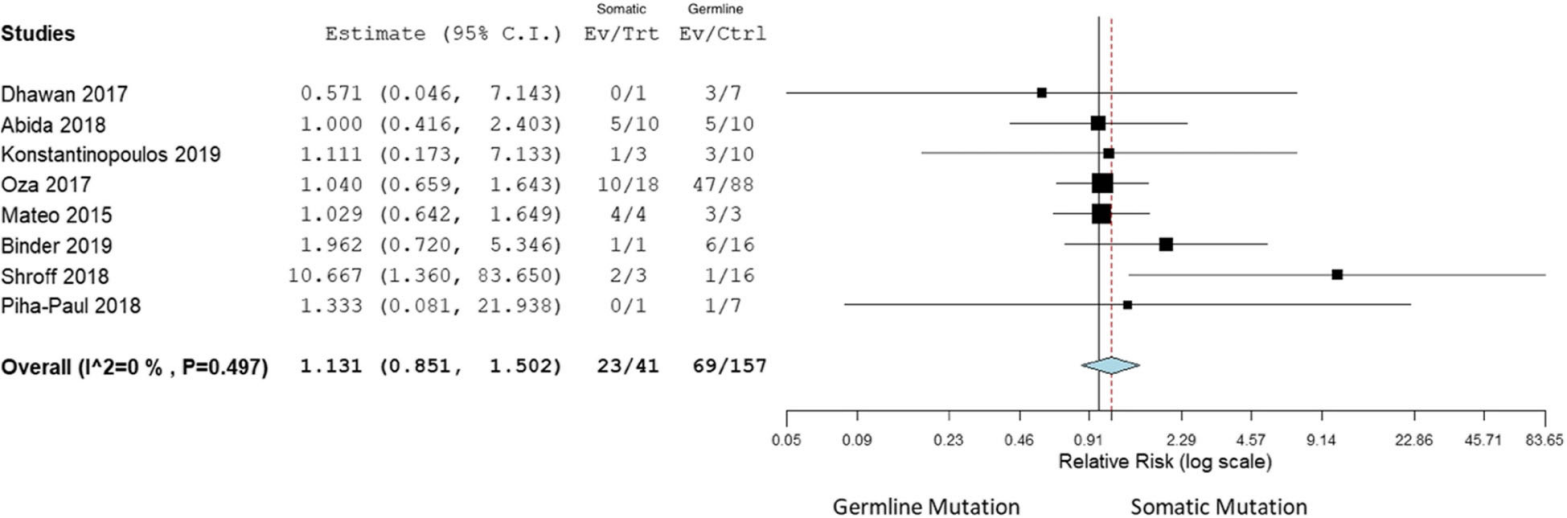
Forest plot representing comparison of response rate between somatic versus germline BRCA mutations (CI: Confidence interval)

Within the eight studies for the patients for which ORR was clearly evaluated, 43 patients with somatic BRCA mutations received PARPi, and 157 patients with germline BRCA patients received PARPi. 24 out of 43 patients with somatic BRCA mutations (55.8%), and 69 out of 157 (43.9%) patients with germline BRCA patients had a response to therapy to PARPi. This difference was not statistically significant (pooled OR 1.13 with 95%CI 0.85–1.49, *p* value = 0.399, I2 = 0) (Fig. 2).

Subgroup analysis was done to determine any difference in ORR amongst different groups listed below between somatic versus germline BRCA.

#### Cancer type

Amongst the eight studies that reported ORR, 2 studies each were exclusively for prostate cancer, ovarian cancer and pancreatic cancer, whereas 2 studies recruited patients with various malignancies.

Amongst the two studies for prostate cancer (Abida et al.^31^ and Mateo et al.^26^), the pooled response was 10/16 for somatic BRCA patients (62.5%), and 8/13 (61.5%) for germline BRCA patients (*p* = 0.92).

Amongst the two studies for pancreatic cancer (Binder et al.^28^ and Shroff et al.^29^), the pooled response was 3/4 for somatic BRCA patients (75%) and 7/32 (21.9%) for germline BRCA patients, with the numerically increased response rate in somatic BRCA patients not statistically significant (*p* = 0.12).

Amongst the two studies for ovarian cancer (Konstantinopaulos et al. [22] and Oza et al.^30^), the pooled response rate was 11/22 for somatic BRCA patients (50%) and 50/98 (51%) for germline BRCA patients (*p* = 0.84).

#### Type of PARPi

Amongst the eight studies that reported ORR, 4 studies evaluated rucaparib and 2 studies evaluated olaparib, with 2 studies evaluating talazoparib. As the 2 studies that evaluated talazoparib had only 1 somatic BRCA patient each, a further subset analysis for talazoparib was not conducted [35, 36].

Amongst the 4 studies using rucaparib, the pooled response rate was 19/34 (55.9%) for somatic BRCA patients and 59/130 (45.4%) for germline BRCA patients (*p* = 0.27).

Amongst the 2 studies evaluating olaparib, the pooled response rate was 5/7 (71.4%) for somatic BRCA patients and 6/13 (46.1%) for germline BRCA patients (*p* = 0.88).

### Combination with other agents versus PARPi monotherapy

As other agents used with PARPi could influence response, we also assessed for PARPi monotherapy studies versus PARPi combination studies.

Amongst the 6 studies that used PARPi as monotherapy, the pooled response rate was 23/39 (58.9%) for somatic BRCA patients and 63/140 (45%) for germline BRCA patients (*p* = 0.35).

Amongst the 2 studies that used PARPi in combination with other agents, the pooled response rate was 1/4 (25%) for somatic BRCA patients and 6/17 (35.3%) for germline BRCA patients (p = 0.35).

### Publication Bias

**Fig. 3:**
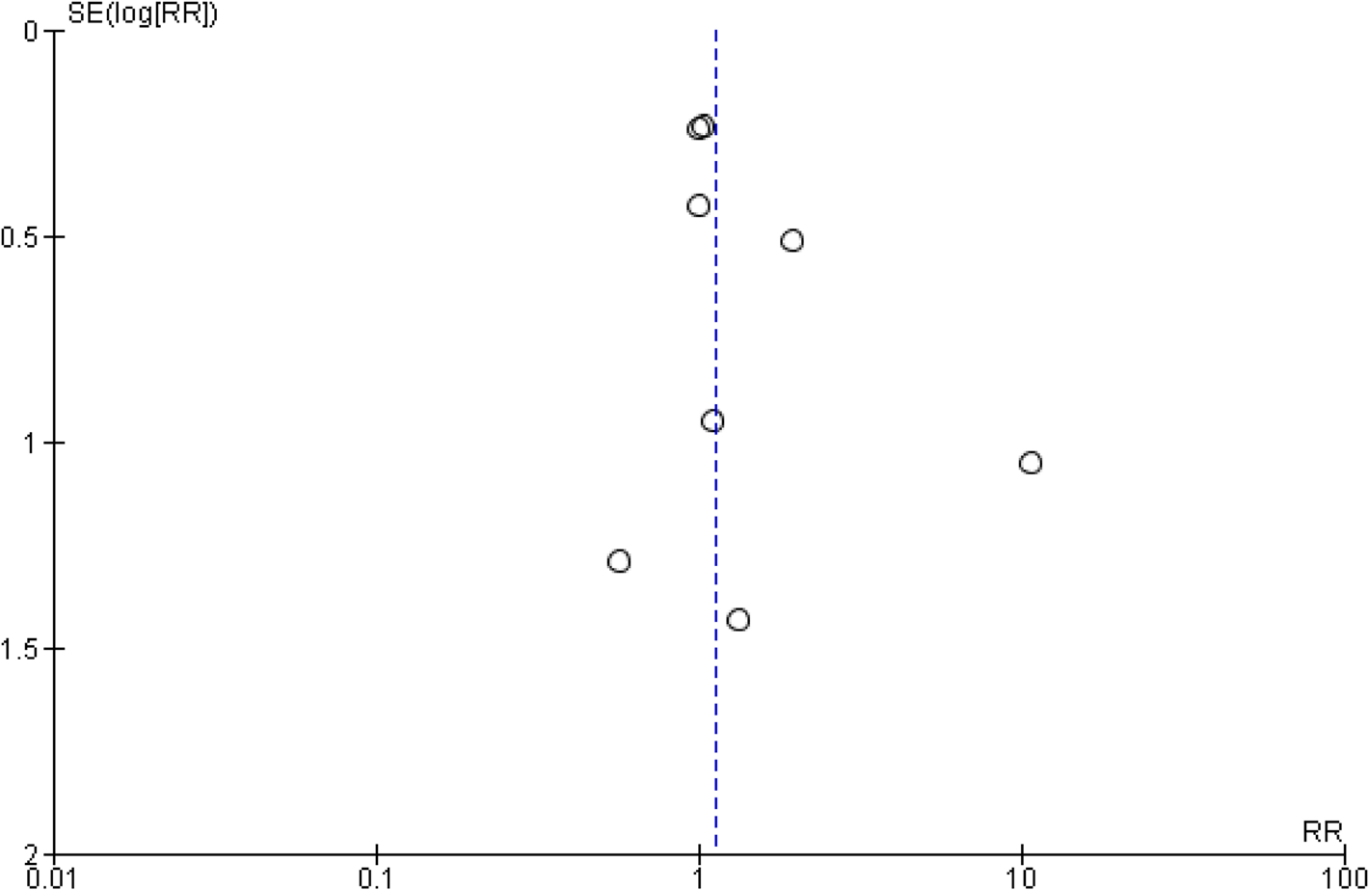
Funnel plot showing visible asymmetry for published studies

Funnel plot represented below (Fig. 3) represented visible asymmetry for published studies signifying a significant publication bias. Supplementary Table S2 highlights risk of bias for each study.

#### Progression-free survival data

Only a total of five studies clearly described PFS data for both somatic and germline BRCA patients (Table 2). This amounted to a total of 111 patients with somatic mutations and 569 patients with germline BRCA mutations.

**Table 2:** Progression-Free Survival (PFS) data for somatic versus germline BRCA mutations

Study Name	PFS for somatic BRCA	PFS for germline BRCA	Statistical difference between somatic versus germline
ARIEL3 [21]	HR of 0.23 (0.10–0.54) compared to placebo, Median PFS 15.7 months	HR of 0.25 (0.16–0.39 germline) compared to placebo, Median PFS 24 months	Not provided
ENGOT 0 V16/NOVA [33]	HR of 0.27 compared to placebo	HR of 0.27 compared to placebo	Not provided
STUDY 19 [23]	HR of 0.23 (0.04 to 1.12) versus placebo, 3/10 progression events	HR of 0.17 (0.09 to 0.34) versus placebo, 16/49 progression events	Not provided
V. Rodriguez-Freixinos et al. [37]	Absolute value not reported	Absolute value not reported	HR of 0.75 for PFS (0.4–1.41) between somatic versus germline, *p* = 0.38
Labidy-Galy et al. [27]	6.8 months (5.1-NA) Median PFS	16.3 (10.4–19.8) median PFS	HR of 1.4 (0.5–3.9), *p* = 0.52

The PFS data was presented in a heterogenous and non-uniform way limiting a meta-analysis. Table 2 indicates PFS data for the five studies. In all five studies, there was no obvious difference in outcomes between somatic versus germline BRCA patients, however a precise statistical analysis could not be performed.

## Discussion

The PARPi are a novel drug group that inhibit the activity of PARP1, PARP2 and PARP3, a group of proteins closely involved in the repair of single strand DNA strand breaks [11]. When these molecules are inhibited, the BRCA-mutated cells are unable to undergo the significant homologous recombination or DNA strand repair needed to fix the errors, effectively leading to arrest during the cell replication cycle, and eventually to apoptosis [12, 13]. With further characterization of BRCA mutations and development of more PARPi, therapeutic options for individuals with advanced ovarian cancers as well as certain breast cancers are increasing, with expanded use of these drugs currently undergoing investigation [14, 38]. Olaparib subsequently gained approval in metastatic breast cancer with germline BRCA mutations based on the OlympiAD trial in 2017 [38], niraparib gained approval as maintenance treatment for patients with ovarian cancer who are responding to platinum-based chemotherapy in 2017 based on the NOVA trial [33], and talazoparib was also approved in 2018 for advanced breast cancer with germline BRCA mutations [39].

Germline mutations of BRCA1 and BRCA2 are significantly associated with the development of multiple neoplasms, including breast, ovarian, stomach, pancreas, colon, and melanoma [3–5]. However somatic BRCA mutations are under-recognized and represent a missed opportunity for further targeted therapy [6]. Many of the existing clinical trials for the PARPi don’t specifically include somatic BRCA patients, leading to underrepresentation in the data. The initial clinical trial, Study 42, leading to FDA approval for olaparib in advanced ovarian cancer, only examined germline BRCA mutations [40]. The OlympiAD phase III trial for olaparib in breast cancer by Robson et al. required a deleterious or suspected deleterious germline BRCA mutation for eligibility [38]. The phase III trial for talazoparib in advanced breast cancer by Litton et al. included only germline mutation in BRCA1/2 as well [39]. The recent POLO trial for pancreatic cancer which studied olaparib as maintenance therapy also did not include any somatic BRCA patients [41].

Exclusion of such patients from clinical trials has thus led to a lack of data of efficacy of PARPi in this subgroup. Furthermore, given concerns over whether somatic BRCA mutations are passenger or driver mutations, further data is needed to establish efficacy in this setting [15]. A potential expansion of the indication of approved medications for germline BRCA to somatic BRCA can provide care to a greater number of patients [6, 8–10].

Our data demonstrates a comparable ORR of PARPi in somatic and germline BRCA patients. A subgroup analyses accounting for different malignancy types and different PARPi also did not reveal any significant difference in response rates. Although we were unable to perform a meta-analysis of PFS data, the reviewed literature indicates similar efficacy of PARPi between somatic versus germline BRCA in terms of PFS. Our data indicates that broader testing for somatic BRCA mutations should be considered.

Our study was limited by inconsistent reporting of PFS and ORR. The heterogeneity of the setting of use for PARPi in the studies included may limit generalizability as well, as in some studies the PARPi was used for maintenance therapy and in others for later lines of treatment.

Furthermore, we could not include several significant publications in our analysis due to exclusion of either somatic of germline BRCA patients, or no clear reporting of outcome specifically for each subgroup. For example, the POLO trial studying olaparib for pancreatic cancer did not include somatic BRCA patients and could not be included [41]. Conversely, the PROFOUND trial in prostate cancer which is looking at the efficacy of PARPi in patients with an array of somatic mutations involved in homologous recombination repair pathways does not include any germline mutation patients, and could not be included in our study [42]. Some studies are known to have included both somatic and germline patients, but do to a lack of subset analysis reported specifically for somatic BRCA patients, they could not be included for analysis, such as the QUADRA study [32].

Our study is the first to review all currently published data to compare ORR between somatic BRCA mutations and germline BRCA mutations when treated with a PARPi. Our analyses revealed a similar ORR between both cohorts. Our data would suggest that broader indications for PARPi therapy may be considered. Furthermore, our data supports the inclusion of somatic BRCA patients in future clinical trials for PARPi therapy. An example of such a study is the NIRAPANC trial enrolling metastatic pancreatic cancer patients with somatic and germline BRCA mutations, as well as other DNA repair deficiencies [43]. Notably in the COVID-19 era, PARP inhibitors, due to its per oral administration, has the added advantage of limiting patient in person healthcare visits and associated exposure, for instance compared to intravenously administered anti-cancer agents, especially with the availability of telemedicine follow-up.

## Conclusion

The use of PARPi in BRCA-related malignancy have largely been limited to BRCA germline mutations, and thus a precise estimate of efficacy of PARPi in somatic BRCA mutation in lacking. Our meta-analysis and systematic review of the literature indicates similar response rates of PARPi therapy in patients with somatic and germline BRCA mutations. Investigation of use of PARPi therapy in a broader patient population, and the inclusion of patients with somatic BRCA mutations in further clinical trials is paramount in improving therapeutic options for our patients.

## Supplementary information

**Additional file 1: Table S1.** Embase search strategy

**Additional file 2: Table S2.** Assessment of bias risk in each study

## Data Availability

The datasets used and/or analyzed during the current study are available from the corresponding author on reasonable request. The studies from which the data was generated are publicly available.

## Abbreviations

PARP: poly(ADP-ribose) polymerase
PARPi: poly(ADP-ribose) polymerase inhibitors
PFS: progression-free survival
ORR: overall response rate
RCT: randomized controlled trials
CI: confidence interval
OR: odds ratio
