# Supplementary material for: Similar response rates and survival with PARP inhibitors for patients with solid tumors harboring somatic versus Germline BRCA mutations: a Meta-analysis and systematic review
Source: BMC Cancer. 2020 Jun 3;20:507. doi: 10.1186/s12885-020-06948-5 (PMC7267765; doi:10.1186/s12885-020-06948-5)
Supplement: Supplementary file 1 — Additional file 1: Table S1. Embase search strategy [file 12885_2020_6948_MOESM1_ESM.docx]

Supplementary table 1: Embase search strategy

| No. | Query | Results |
| --- | --- | --- |
| #8 | #6 AND #7 | 136 |
| #7 | 'clinical trial'/exp | 1430515 |
| #6 | #5 NOT ('chapter'/it OR 'conference review'/it OR 'editorial'/it OR 'note'/it OR 'review'/it OR 'short survey'/it) | 496 |
| #5 | #3 AND #4 | 640 |
| #4 | #1 AND #2 | 4418 |
| #3 | somatic OR 'non germline' OR 'non germ line' OR 'soma cell*' OR 'somatic cell'/exp | 156097 |
| #2 | brca* | 38770 |
| #1 | 'nicotinamide adenine dinucleotide adenosine diphosphate ribosyltransferase inhibitor'/exp OR parpi OR 'nicotinamide adenine dinucleotide adenosine diphosphate ribosyltransferase inhibit*' OR 'nad adp ribosyltransferase inhibit*' OR 'parp inhibit*' OR 'pars inhibit*' OR 'poly adp ribose synthetase inhibit*' OR 'poly adp ribose polymerase inhibit*' OR 'poly adp ribosylation inhibit*' OR '1,5 dihydroisoquinoline' OR '1,5 dihydroxyisoquinoline' OR '3 aminobenzamide' OR '3 methoxybenzamide' OR '3 nitrosobenzamide' OR '4 3 4 cyclopropanecarbonylpiperazine 1 carbonyl 4 fluorobenzyl 2h phthalazin 1 one' OR '4 amino 1,8 naphthalimide' OR '6 amino 1,2 benzopyrone' OR '6 nitroso 1,2 benzopyrone' OR cofpropamine OR 'gpi 15427' OR iniparib OR ku0058948 OR me0328 OR olaparib OR phenanthridone OR rucaparib OR talazoparib OR veliparib OR amelparib OR pamiparib OR niraparib | 13686 |
