# Supplementary material for: Similar response rates and survival with PARP inhibitors for patients with solid tumors harboring somatic versus Germline BRCA mutations: a Meta-analysis and systematic review
Source: BMC Cancer. 2020 Jun 3;20:507. doi: 10.1186/s12885-020-06948-5 (PMC7267765; doi:10.1186/s12885-020-06948-5)
Supplement: Supplementary file 2 — Additional file 2: Table S2. Assessment of bias risk in each study [file 12885_2020_6948_MOESM2_ESM.docx]

Supplementary Table S2: Assessment of bias risk in each study

| Study | Random sequence generation (selection bias) | Allocation concealment (selection bias) | Blinding of participants and personnel (performance bias) | Blinding of outcome assessment (detection bias) | Incomplete outcome data (attrition bias) | Selective reporting (reporting bias) |
| --- | --- | --- | --- | --- | --- | --- |
| Dhawan 2017 | High | High | High | High | NA | Low |
| Abida 2018 | High | High | High | High | NA | Low |
| Konstantinopaulos 2019 | High | High | High | High | NA | Low |
| Oza 2017 | High | High | High | High | NA | Low |
| Mateo 2015 | High | High | High | Low | NA | Low |
| Binder 2019 | High | High | High | High | NA | Low |
| Shroff 2018 | High | High | High | High | NA | Low |
| Piha-Paul 2018 | High | High | High | High | NA | Low |
